# Evidence of Allopolyploidy in *Urochloa humidicola* Based on Cytological Analysis and Genetic Linkage Mapping

**DOI:** 10.1371/journal.pone.0153764

**Published:** 2016-04-22

**Authors:** Bianca B. Z. Vigna, Jean C. S. Santos, Leticia Jungmann, Cacilda B. do Valle, Marcelo Mollinari, Maria M. Pastina, Maria Suely Pagliarini, Antonio A. F. Garcia, Anete P. Souza

**Affiliations:** 1 University of Campinas (UNICAMP), Center of Molecular Biology and Genetic Engineering (CBMEG), CP 6010, CEP 13083–970, Campinas, SP, Brazil; 2 Embrapa Pecuária Sudeste, CP 399, CEP 13560–970, São Carlos, SP, Brazil; 3 Embrapa Gado de Corte, Av. Radio Maia, 830, CEP 79106–550, Campo Grande, MS, Brazil; 4 University of São Paulo, Escola Superior de Agricultura Luiz de Queiroz, Department of Genetics, CP 83, CEP 13400–970, Piracicaba, SP, Brazil; 5 Embrapa Milho e Sorgo, Rod. MG 424, Km 65, CEP 35701–970, Sete Lagoas, MG, Brazil; 6 University of Maringá (UEM), Department of Genetics and Cell Biology, Av. Colombo, 5790, Zona 7, CEP 87020–900, Maringá, PR, Brazil; 7 University of Campinas (UNICAMP), Biology Institute, Department of Plant Biology, CP6109, CEP 13083–970, Campinas, SP, Brazil; Nanjing Forestry University, CHINA

## Abstract

The African species *Urochloa humidicola* (Rendle) Morrone & Zuloaga (syn. *Brachiaria humidicola* (Rendle) Schweick.) is an important perennial forage grass found throughout the tropics. This species is polyploid, ranging from tetra to nonaploid, and apomictic, which makes genetic studies challenging; therefore, the number of currently available genetic resources is limited. The genomic architecture and evolution of *U*. *humidicola* and the molecular markers linked to apomixis were investigated in a full-sib F_1_ population obtained by crossing the sexual accession H031 and the apomictic cultivar *U*. *humidicola* cv. BRS Tupi, both of which are hexaploid. A simple sequence repeat (SSR)-based linkage map was constructed for the species from 102 polymorphic and specific SSR markers based on simplex and double-simplex markers. The map consisted of 49 linkage groups (LGs) and had a total length of 1702.82 cM, with 89 microsatellite loci and an average map density of 10.6 cM. Eight homology groups (HGs) were formed, comprising 22 LGs, and the other LGs remained ungrouped. The locus that controls apospory (apo-locus) was mapped in LG02 and was located 19.4 cM from the locus Bh027.c.D2. In the cytological analyses of some hybrids, bi- to hexavalents at diakinesis were observed, as well as two nucleoli in some meiocytes, smaller chromosomes with preferential allocation within the first metaphase plate and asynchronous chromosome migration to the poles during anaphase. The linkage map and the meiocyte analyses confirm previous reports of hybridization and suggest an allopolyploid origin of the hexaploid *U*. *humidicola*. This is the first linkage map of an *Urochloa* species, and it will be useful for future quantitative trait locus (QTL) analysis after saturation of the map and for genome assembly and evolutionary studies in *Urochloa* spp. Moreover, the results of the apomixis mapping are consistent with previous reports and confirm the need for additional studies to search for a co-segregating marker.

## Introduction

The African species *Urochloa humidicola* (Rendle) Morrone & Zuloaga (syn. *Brachiaria humidicola* (Rendle) Schweick.), also known as koronivia grass, is an important perennial grass of African origin that is used throughout the tropics as a pasture grass, including in Central and South America, Southeast Asia and Oceania [1]. This species reproduces through facultative pseudogamic and aposporous apomixis [2], but a sexual genotype has been identified [3]. Genetic and molecular studies of the genus have demonstrated that apospory is controlled by a single dominant locus [4, 5, 6]. The basic chromosome number of *U*. *humidicola* has been reported as x = 6 [7, 8, 9, 10], and its number of chromosomes ranges from 36 to 54 [11]. The species has a DNA content of approximately 1953 Mbp/1C or 651 Mbp/1Cx [12], and it contains the largest chromosomes among the five *Urochloa* species [13]. Cytogenetic and molecular studies of different *Urochloa* species suggest that these species underwent hybridization between two distinct genomes [10, 14], allopolyploidization [15, 16, 17] and the formation of non-reduced gametes [18]. However, the origin of the polyploidy of the genus remains unknown.

The mapping process is more complex in polyploids than in diploids because of the larger number of possible genotypes, the difficulties associated with the identification of these genotypes, and the limited knowledge regarding the type of polyploidy in many species [19]. A method based on the segregation analysis of single-dose markers (SDMs) has been developed [20]; in this method, an SDM is present in only one of the parents of the cross, with a 1:1 segregation ratio, regardless of the level and type of ploidy. SDMs in both parents segregate in a 3:1 ratio and can also be used for genetic mapping, thereby providing important information for the integration of maps of the parents that are obtained when only 1:1 loci are used. Thus, mapping in polyploids is conducted in two steps: (1) ordination of the loci in individual linkage groups (LGs) and subsequent integration of the maps, and (2) designation of these LGs into homology groups (HGs) [19]. Putative HGs can be identified using markers that recognize various SDMs within the same loci [21] through multiple-dose markers (MDMs) [19], through the identification of (at least) two probes common to two linkage groups [22], or through the detection of linkage in the repulsion phase between markers located in homologous chromosomes [23]. Genetic maps of polyploid species based on SDMs have been developed for various grass species, such as *Poa pratensis* [24], *Pennisetum ciliare* [25], *Festuca arundinacea* [26], *Panicum maximum* [27], *Cynodon sp*. [28], *Paspalum notatum* [29], *Saccharum spp*. [30, 31] and *Panicum virgatum* [32].

In addition to polyploidy, apomixis also interferes with mapping because it does not allow the production of inbred lines. Thus, mapping should be conducted in a full-sib family that originates from heterozygous parents, for which many loci segregate and the linkage phase of the loci is usually unknown [33].

Genetic mapping of apospory has been performed for the tropical forage grasses *Panicum maximum* [27] and *Paspalum notatum* [29]; in both cases, apospory has been mapped to a single linkage group. Both the grasses *Pennisetum squamulatum* and *Cenchrus ciliaris* have been studied in detail, and an *Apospory-Specific Genomic Region* (ASGR), which is conserved and does not exhibit recombination, has been described [34, 35, 36, 37, 38, 39, 40]. However, there have been no extensive studies of the *Urochloa* genus. Two studies described bulk segregant analyses in interspecific hybrids between *U*. *brizantha* and *U*. *ruziziensis* and mapped the apo-locus region with RFLP, RAPD and AFLP markers [5, 6]. For *U*. *humidicola*, [41] described an RAPD marker located at a distance of 4.6 cM from the apo-locus using a bulk segregant analysis of 100 intraspecific hybrids.

Previous studies have used molecular markers as tools for the genetic breeding of *U*. *humidicola*. The genetic diversity of the koronivia grass preserved in its germplasm bank has been described using RAPD [42] and microsatellite markers [43]. However, no linkage map has been developed for the species.

The objective of this study was to obtain a better understanding of the polyploidy and transmission genetics of koronivia grass, to provide useful DNA markers for forage breeding programs through the construction of a framework *U*. *humidicola* linkage map and to map the apo-locus. To achieve these goals, microsatellite markers were used, and the map was built using a multipoint approach; chromosome association analyses of the hybrid meiocytes were performed. The plant material studied was the full-sib progeny of 279 F_1_ hybrids derived from an intraspecific cross between the non-inbred and heterozygous parents BRA005811 (hereafter referred to as H031), a sexual accession, and the apomictic cultivar *U*. *humidicola* cv. BRS Tupi (pollen donor), both of which are hexaploid (2n = 6x = 36) [44].

## Results

### Meiotic Analysis

**Fig 1:**
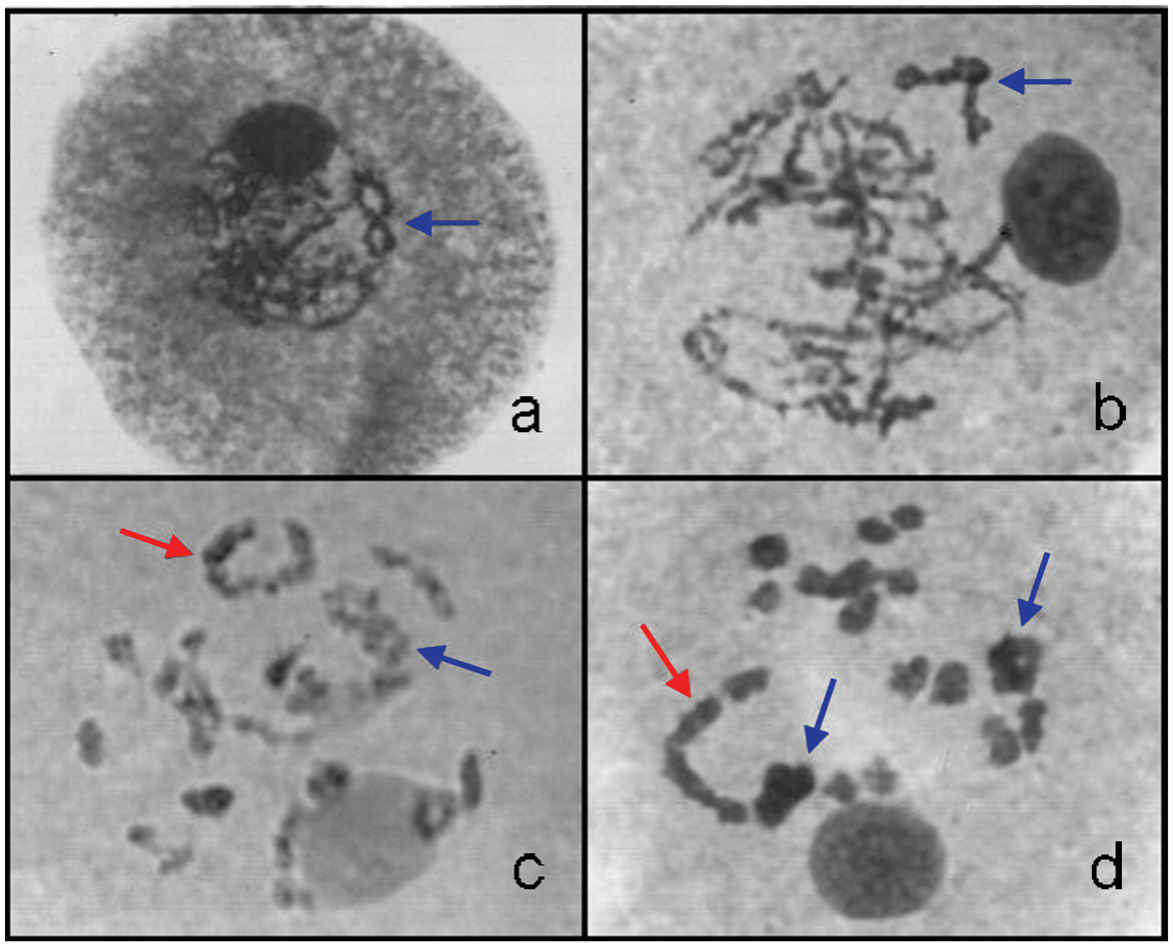
Meiocytes in the *U*. *humidicola* hybrids (2n = 36) exhibiting multiple-chromosome associations at prophase I. a) Meiocyte in pachytene exhibiting a tetravalent (blue arrow). b) Meiocyte in diplotene exhibiting a tetravalent (blue arrow). c, d) Meiocytes in diakinesis exhibiting tetra- and hexavalents (blue and red arrows, respectively) with bivalents already in separation.

The chromosome associations ranged from only bivalents to mixtures of bi- and tetravalents, bi- and hexavalents and bi-, tetra- and hexavalents. There was a predominance of 16 bi- and one tetravalent, followed by 14 bi- and two tetravalents. Hexavalents were recorded at a low frequency (Table 1). Fig 1a and 1b show a meiocyte in pachytene with a tetravalent association, respectively, and Fig 1c and 1d show a meiocyte in diakinesis with tetra- and hexavalent associations, respectively.

**Table 1:** Percentage of chromosome associations at diakinesis in the genitors and hybrids scored among 20 meiocytes per genotype.

Genotype	Chromosome associations at diakinesis (% of meiocytes)										
	18 II	16 II + 1 IV	14 II + 2 IV	12 II + 3 IV	10 II + 4 IV	8 II + 5 IV	15 II + 1 VI	12 II + 2 VI	13 II + 1 IV + 1 VI	11 II + 2 IV + 1 VI	9 II + 3 IV + 1 VI
H031	50.0	20.0	10.0	-	-	-	20.0	-	-	-	-
cv. BRS Tupi	20.0	20.0	20.0	10.0	-	5.0	25.0	-	-	-	-
Hb 01	-	15.0	45.0	40.0	-	-	-	-	-	-	-
Hb 02	20.0	20.0	5.0	20.0			5.0	5.0	5.0	10.0	10.0
Hb 03	-	-	-	-	-	-	-	-	-	-	-
Hb 07	30.0	40.0	20.0	10.0	-	-	-	-	-	-	
Hb 08	50.0	30.0	5.0	-	-	-	10.0	-	5.0	-	-
Hb 11	65.0	25.0	10.0	-	-	-	-	-	-	-	-
Hb 12	20.0	30.0	40.0	10.0	-	-	-	-	-	-	-
Hb 16	-	-	-	-	-	-	-	-	-	-	-
Hb 24	-	65.0	25.0	10.0	-	-	-	-	-	-	-
Hb 29	20.0	45.0	30.0	5.0	-	-	-	-	-	-	-
Hb 30	10.0	40.0	25.0	-	-	-	-	25.0	-	-	-
Hb 36	-	45.0	30.0	25.0	-	-	-	-	-	-	-
Hb 45	-	30.0	60.0	10.0	-	-	-	-	-	-	-
Hb 54	-	20.0	35.0	20.0	-	-	-	-	-	-	-
Hb 76	15.0	25.0	15.0	40.0	-	-	5.0	-	-	-	-
Hb 83	-	55.0	40.0	5.0	-	-	-	-	-	-	-
Hb 84	-	85.0	15.0	-	-	-	-	-	-	-	-
Hb 88	-	-	-	-	-	-	-	-	-	-	-
Hb 100	-	5.0	40.0	40.0	15.0	-	-	-	-	-	-
Hb 101	10.0	25.0	15.0	25.0	20.0	-	-	-	-	-	-
Hb 111	-	-	-	-	-	-	-	-	-	-	-
Hb 115	-	45.0	55.0	-	-	-	5.0	-	-	-	-
Hb 117	10.0	70.0	20.0	-	-	-	-	-	-	-	-
Hb 120	40.0	20.0	40.0	-	-	-	-	-	-	-	-
Hb 136	20.0	60.0	20.0	-	-	-	-	-	-	-	-
Hb 146	20.0	10.0	-	-	20.0	-	10.0	-	40.0	-	-
Hb 151	20.0	45.0	25.0	10.0	-	-	-	-	-	-	-
Hb 153	40.0	40.0	10.0	-	-	-	10.0	-	-	-	-
Hb 176	15.0	50.0	35.0	-	-	-	-	-	-	-	-
Hb 179	25.0	50.0	15.0	10.0	-	-	-	-	-	-	-
Hb 185	10.0	60.0	30.0	-	-	-	-	-	-	-	-
Hb 193	10.0	50.0	30.0	-	-	-	-	10.0	-	-	-
Hb 196	5.0	60.0	20.0	10.0	-	-	10.0	-	-	-	-
Hb 216	15.0	50.0	15.0	20.0	-	-	-	-	-	-	-
Hb 227	25.0	35.0	30.0	-	-	-	10.0	-	-	-	-
Hb 242	35.0	55.0	10.0	-	-	-	-	-	-	-	-
Hb 244	20.0	45.0	30.0	-	-	-	5.0	-	-	-	-
Hb 267	35.0	10.0	25.0	15.0	-	-	5.0	-	5.0	5.0	-
Hb 269	20.0	50.0	30.0	-	-	-	-	-	-	-	-
Hb 270	15.0	45.0	40.0	-	-	-	-	-	-	-	-
Hb 289	5.0	55.0	35.0	-	-	-	5.0	-	-	-	-
Hb 297	15.0	20.0	40.0	20.0	-	-	-	-	-	-	5.0
Hb 343	-	-	-	-	-	-	-	-	-	-	-
Hb 347	5.0	50.0	20.0	20.0	-	-	5.0	-	-	-	-
Hb 350	15.0	45.0	30.0	-	-	-	5.0	5.0	-	-	-

**Fig 2:**
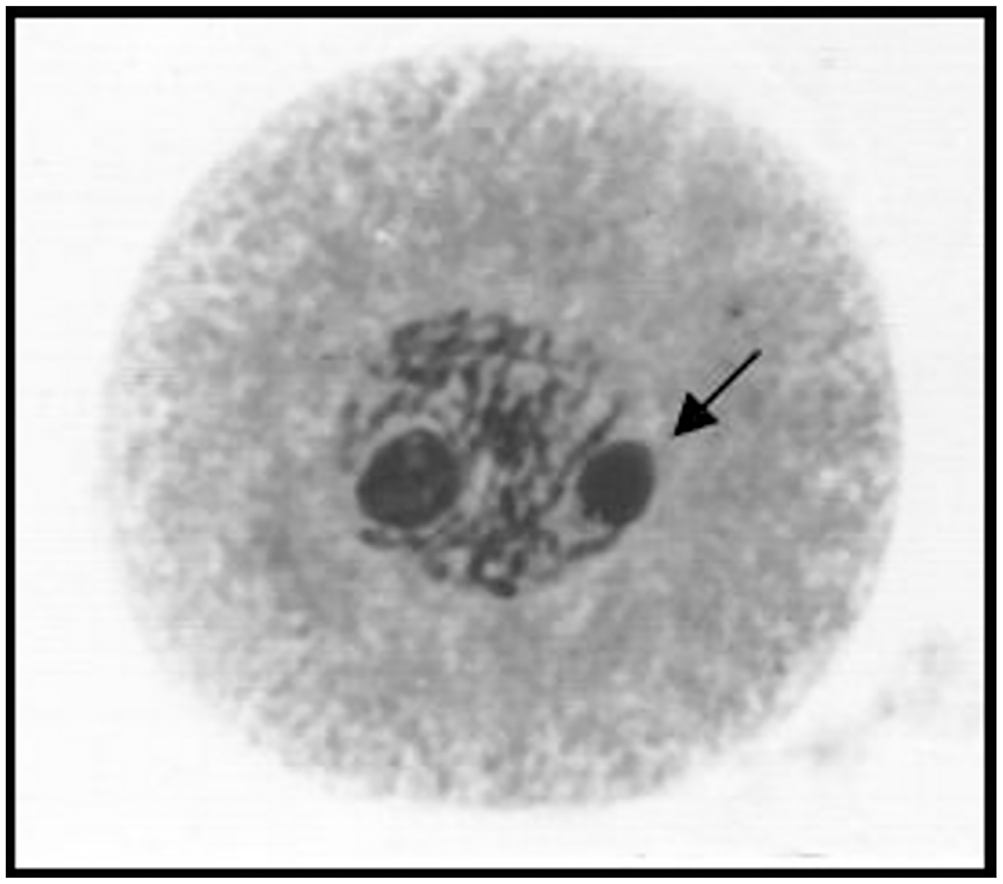
Meiocyte in pachytene exhibiting two nucleoli of different sizes. Meiocyte in pachytene exhibiting two nucleoli of different sizes. The arrow indicates the smaller nucleolus.

**Fig 3:**
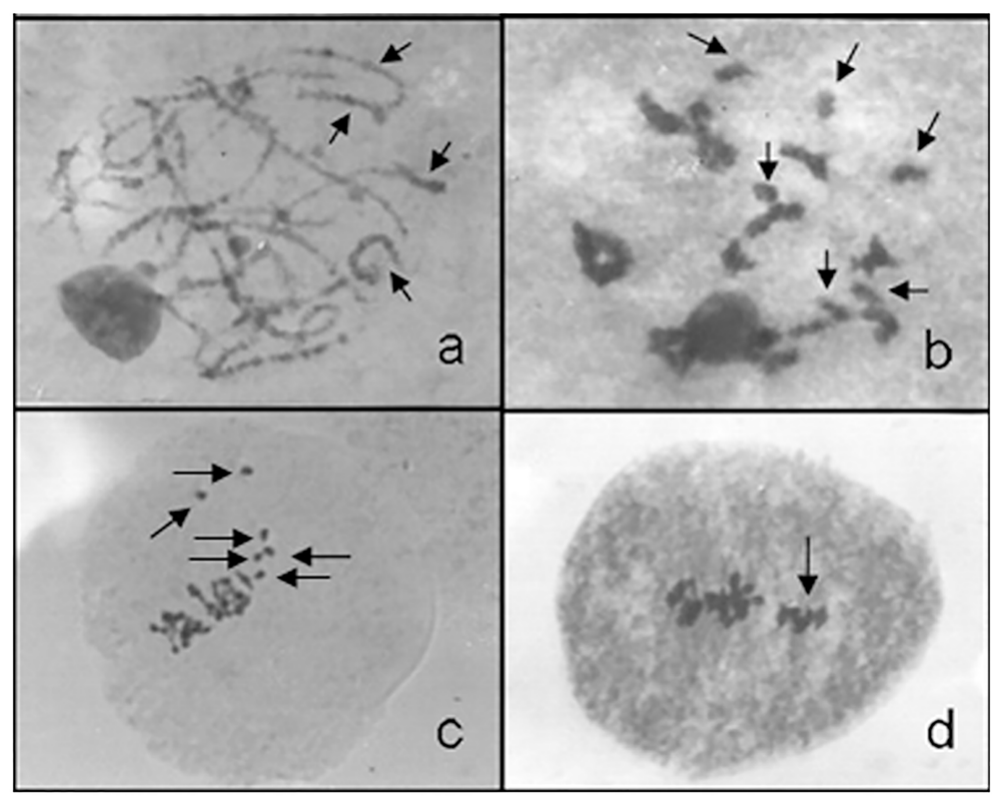
Meiocyte in pachytene exhibiting small chromosomes. a) Meiocyte in pachytene exhibiting several small chromosomes (arrows). b) Meiocyte in diakinesis exhibiting six small bivalents (arrows). c) Metaphase I with the six small bivalents allocated peripherally in the plate (arrow). d) Metaphase I with the six small bivalents separated in the metaphase plate. The arrow indicates the set of six small bivalents.

The mean percentage of meiotic abnormalities at diakinesis among the 45 evaluated hybrids ranged from 20.72 to 81.40% (Table 2), based on a previous study [44]. The presence of two nucleoli was observed in some meiocytes of the *U*. *humidicola* hybrids (Fig 2). The cytological analyses of the meiocytes at pachytene in the hybrids revealed chromosomes with different sizes and heterochromatic regions and a preferential allocation of the small chromosomes within the metaphase plate (Fig 3).

**Table 2:** Percentage of meiotic abnormalities in the genitors and hybrids of *Urochloa humidicola*, based on [44].

Genotype	Mode of reproduction	N^o^ of PMCs	Mean of abnormalities (%)
H031	Sexual	1492	32.35
cv. BRS Tupi	Apomictic	2403	9.50
Hb 01	Apomictic	498	20.72
Hb 02	Sexual	607	43.04
Hb 03	Apomictic	908	73.63
Hb 07	Apomictic	724	24.95
Hb 08	Apomictic	458	45.41
Hb 11	Sexual	573	63.16
Hb 12	Apomictic	426	38.92
Hb 16	Sexual	535	44.22
Hb 24	Sexual	751	46.62
Hb 29	Apomictic	424	43.39
Hb 30	Apomictic	583	78.02
Hb 36	Apomictic	436	48.38
Hb 45	Sexual	451	51.15
Hb 54	Apomictic	453	40.99
Hb 76	Sexual	471	55.00
Hb 83	Apomictic	423	46.94
Hb 84	Sexual	437	33.49
Hb 88	Apomictic	1131	61.51
Hb 100	Apomictic	461	56.69
Hb 101	Apomictic	474	69.05
Hb 111	Apomictic	526	85.37
Hb 115	Apomictic	520	48.00
Hb 117	Sexual	597	58.37
Hb 120	Apomictic	884	58.74
Hb 136	Apomictic	511	61.73
Hb 146	Apomictic	898	68.67
Hb 151	Sexual	493	55.26
Hb 153	Apomictic	576	67.40
Hb 176	Apomictic	482	60.87
Hb 179	Apomictic	474	75.70
Hb 185	Apomictic	900	55.50
Hb 193	Apomictic	939	69.78
Hb 196	Sexual	696	63.26
Hb 216	Sexual	970	47.85
Hb 227	Apomictic	511	62.77
Hb 242	Apomictic	492	45.52
Hb 244	Sexual	735	57.47
Hb 267	Apomictic	550	73.33
Hb 269	Apomictic	526	62.53
Hb 270	Apomictic	559	74.43
Hb 289	Sexual	1088	74.81
Hb 297	Apomictic	633	81.40
Hb 343	Sexual	370	46.85
Hb 347	Apomictic	503	74.01
Hb 350	Sexual	797	64.15

### Microsatellite markers

Sixty-six new primer pairs were designed and amplified successfully in *U*. *humidicola* (BhUNICAMP140-BhUNICAMP205), and 54 were polymorphic among the parents of the cross and six hybrids. Between one and 10 bands were observed per locus, with a mean of 4.06 bands per locus (S1 Table). The polymorphism information content (PIC) values and discrimination power (DP) of each locus were not determined because of the small number of genotypes evaluated.

Among all of the microsatellite loci developed for the species, 124 were selected for mapping (S1 and S2 Tables), which resulted in 22 monomorphic loci and 102 polymorphic loci. In total, 479 bands were amplified (Table 3, S3 Table), with a mean of 4.7 bands per locus.

**Table 3:** Summary of the SSR loci used in the genetic linkage map construction.

	SSR	Total bands	Linked bands
Loci selected for mapping	124	-	-
Monomorphic loci	22	-	-
Polymorphic loci	102	479	-
Single-dose markers (1:1)	-	194	129
D1.13 (ao x oo)	-	80	56
D2.18 (oo x ao)	-	114	83
Double-simplex markers (3:1) C8 (ao x ao)	-	33	22
Total (1:1 and 3:1)	89	227	161
Number of markers with segregation distortion	13	252	-

We made several observations regarding the amplification profiles of the loci. Two sets of bands were observed in 50.6% of the loci; one set contained more bands than the other. The sexual parent (H031) did not amplify in eight loci (S1 Fig).

### Linkage analysis

Almost half (227 out of 479 amplified bands) of the genotyped bands presented the expected segregation ratios for single-dose markers and were mapped (Table 3). Forty percent of the amplified bands were polymorphic between the parents of the cross and presented a 1:1 segregation pattern, which was expected for the single-dose bands in the mapping population (type D bands), whereas 33 bands (6.9%) were monomorphic between the parents of the cross (type C bands) and presented a 3:1 segregation ratio in the mapping population (Table 3), totaling 227 bands that were segregated according to the expected ratios. Among the type D bands, the type D2 bands (with the configuration “oo x ao”, which corresponds to single-dose bands from the male parent) were the most frequent (58.7%), and the type D1 bands (with the configuration “ao x oo”) corresponded to 41.2% of this class of bands. The bands that did not segregate according to the expected ratios corresponded to 52.6% of the total bands evaluated. These bands were considered to exhibit segregation distortion and/or a higher allele dosage and thus could not be used for linkage map construction.

**Fig 4:**
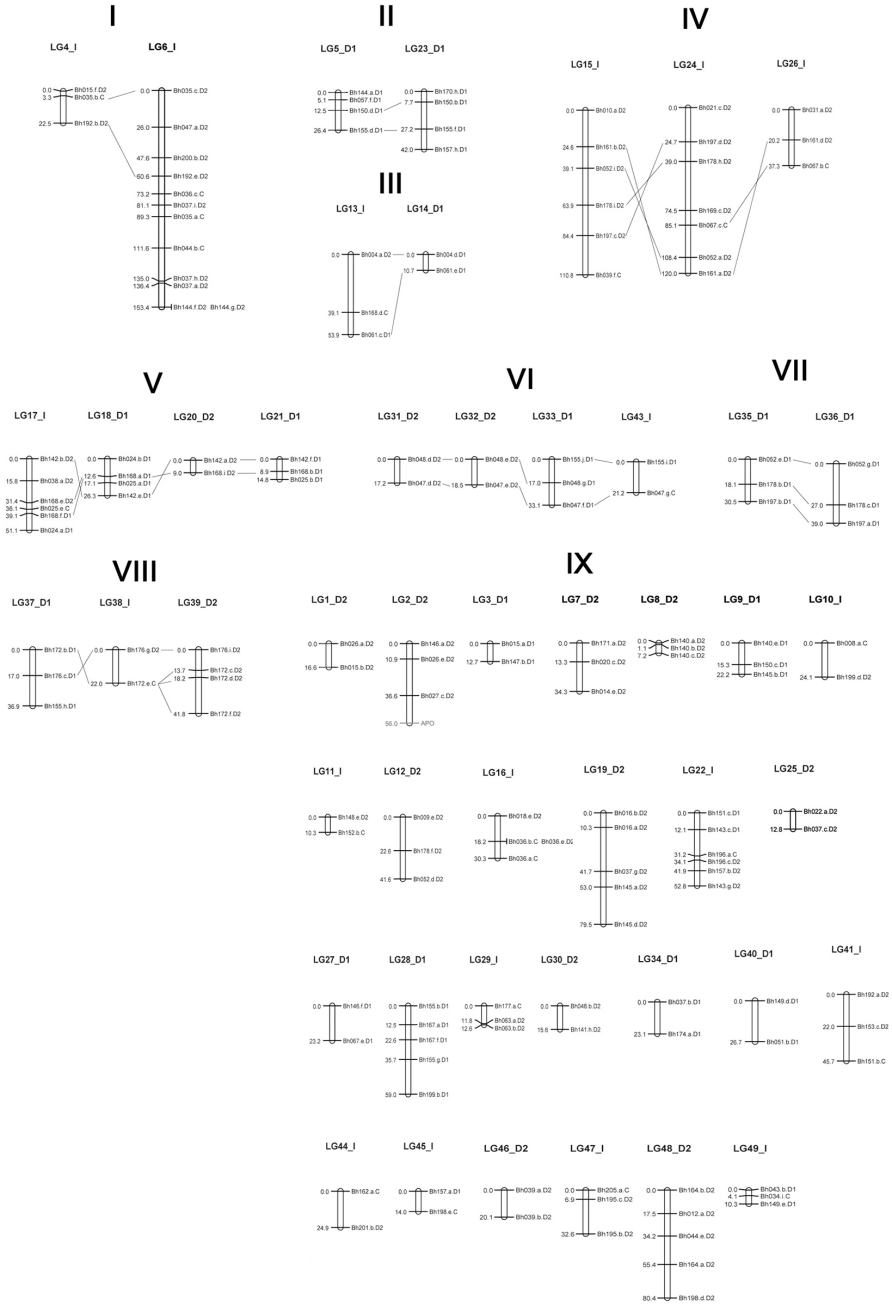
A genetic linkage map for the hexaploid *U*. *humidicola* based on single-dose markers.

Of the 227 bands that segregated according to the expected ratios, 161 were mapped (S1 Table) to 49 LGs with a logarithm of the odds (LOD) = 5. One of these bands corresponded to the apo-locus, whereas the other 66 bands remained unlinked (Fig 4). The LGs presented 3.3 bands per group on average and ranged in size from 1.08 to 153.35 cM, with an average size of 34.7 cM per LG. The map covered 1702.8 cM, with a mean distance between bands (density) of 10.6 cM. Several of the bands formed clusters in the LGs, and others were distributed sparsely, with gaps of up to 39.1 cM (LG13). The type C bands were mapped in 19 different LGs. Three LGs presented the type C, type D1 and type D2 bands together; 13 LGs presented type C and type D2 bands together; and three LGs presented type C and D1 bands together.

Genetic linkage map for *U*. *humidicola* generated from 279 F_1_ hybrids from the cross between the sexual accession H031 and the apomictic cultivar BRS Tupi based on 161 single-dose markers from 89 microsatellite loci. The linkage groups (LGs) are sequentially numbered and assigned into eight homology groups (HGs, I to VIII). Unassigned LGs are located in group IX. The LGs are identified with a letter that corresponds to the type of LG (I: integrated, D1: LG with only D1 bands, D2: LG with only D2 bands). The distances are indicated in centiMorgans (Kosambi) on the left side. The bands are identified according to the method described in the Methods section and indicated on the right side. The apo-locus is indicated in red. The lines represent the alignment between the LGs from the same HG.

The linkages in repulsion were detected and are described in Table 4. All linkage phases are described in S4 Table.

**Table 4:** Linkages in repulsion identified in the LGs and its correspondent LOD score, in bold text. The linkage phase diagram of each band in both genitors of the mapping population are also shown. The configuration ao x ao indicates a type C band, ao x oo indicates a type D1 band, and oo x ao indicates a type D2 band.

LG	Bands	Position (cM)	Parent 1	Parent 2	LOD Score
6	Bh035.c.D2	0	o | | o	a | | o	
	Bh047.a.D2	25.95	o | | o	a | | o	
	Bh201.b.D2	47.55	o | | o	a | | o	
	Bh193.e.D2	60.56	o | | o	a | | o	
	Bh036.c.C	73.19	a | | o	a | | o	
	Bh037.i.D2	81.07	o | | o	a | | o	
	Bh035.a.C	89.29	a | | o	a | | o	
	**Bh044.b.C**	**111.57**	**a | | o**	**a | | o**	**7.69**
	**Bh037.h.D2**	**134.95**	**o | | o**	**o | | a**	
	Bh037.a.D2	136.39	o | | o	o | | a	
	Bh144.f.D2	153.35	o | | o	o | | a	
	Bh144.g.D2	153.35	o | | o	o | | a	
	log-likelihood:	-1285.663			
10	**Bh008.a.C**	**0**	**a | | o**	**a | | o**	**5.20**
	**Bh200.d.D2**	**24.09**	**o | | o**	**o | | a**	
	log-likelihood:	-306.8086			
19	**Bh016.b.D2**	**0**	**o | | o**	**a | | o**	**42.14**
	**Bh016.a.D2**	**10.30**	**o | | o**	**o | | a**	
	Bh037.g.D2	41.65	o | | o	o | | a	
	Bh145.a.D2	52.95	o | | o	o | | a	
	Bh145.d.D2	79.54	o | | o	o | | a	
	log-likelihood:	-674.62			
29	**Bh177.a.C**	**0**	**a | | o**	**a | | o**	**15.86**
	**Bh063.a.D2**	**11.81**	**o | | o**	**o | | a**	
	**Bh063.b.D2**	**12.57**	**o | | o**	**a | | o**	**74.67**
	log-likelihood:	-320.5515			
46	**Bh039.a.D2**	**0**	**o | | o**	**a | | o**	**24.24**
	**Bh039.b.D2**	**20.14**	**o | | o**	**o | | a**	
	log-likelihood:	-321.2515			
47	Bh206.a.C	0	a | | o	a | | o	
	**Bh196.c.D2**	**6.91**	**o | | o**	**a | | o**	**17.70**
	**Bh196.b.D2**	**32.62**	**o | | o**	**o | | a**	
	log-likelihood:	-434.5707			
48	Bh164.b.D2	0	o | | o	a | | o	
	Bh012.a.D2	17.53	o | | o	a | | a	
	**Bh044.e.D2**	**34.15**	**o | | o**	**a | | o**	**21.95**
	**Bh0164.a.D2**	**55.36**	**o | | o**	**o | | a**	
	Bh199.d.D2	80.40	o | | o	o | | a	
	log-likelihood:	-694.9043			

### Homology groups

Eight HGs comprised 22 LGs, and the other LGs remained ungrouped (Fig 4).

### Apospory mapping

The mode of reproduction of 270 hybrids from the mapping population was determined by previous studies [41, 45, 46], and apospory presented a 1:1 segregation ratio with the chi-square test (X^2^ = 3.793, p ≥ 0.05), according to the expected model for monogenic inheritance. The individual identifications of the mode of reproduction are listed in S5 Table.

The apo-locus was linked to LG02, 19.4 cM from the mark Bh027.c.D2, 45.1 cM from the mark Bh026.e.D2 and 56.0 cM from the mark Bh146.a.D2. This linkage group presented only type D2 bands and contained alleles exclusively from the apomictic parent.

## Discussion

The map reported here is the first to be developed for *U*. *humidicola* and consists of a band corresponding to the apo-locus and 160 SDMs derived from newly and previously published microsatellite markers. The new microsatellite markers represent a significant set of tools that will benefit breeding programs for this species and help to address questions regarding its genome and genetics.

A linkage analysis using SDM has been successfully adopted in outcrossing polyploid mapping when endogamic lines cannot be easily obtained [32, 33, 47]. According to a previous study [20], a 75-hybrid progeny is sufficient for detecting single-dose loci that segregate in the population with a 98% confidence level. The present map was constructed based on SDMs detected in a 279-hybrid progeny.

The map was generated based on a methodology developed previously [48] and expanded for multipoint analysis. It presented 49 linkage groups (LGs) with 161 bands (apo-locus included), with a total length of 1702.82 cM and an average map density of 10.6 cM. Thirty-six LGs were expected; however, the identification of 49 LGs may be a result of the small sample size and/or the exclusive usage of SDM for mapping, thereby resulting in LGs that did not link because there were not sufficient bands to establish linkage relationships between two LGs. Many LGs presented only two bands, and 66 bands (29.1%) did not link to any LG.

Methods for mapping loci in polyploids with higher allele dosages are rare in the literature [49, 50] and are reported mostly for autotetraploid species [51, 52] or based on principles that hinder automation of the analysis [48]. The exclusive usage of single-dose markers contributes to obtaining an incipient genetic map, and such markers are the most useful type of marker for polyploid mapping. Although technologies such as restriction site-associated DNA sequencing (RADSeq) and genotyping-by-sequencing (GBS) for the large-scale identification of SNPs are available and suitable for use in polyploid plants [53, 54, 55, 56, 57, 58], including higher dosage determination, it is important that statistical methods be developed to map these data, considering the doses and different ploidies. Some statistical approaches for autotetraploids have been developed but cannot be applied in a straightforward manner to other ploidies [53]. Moreover, SNPs have been reported to achieve similar accuracy [59] and similar genome-wide heterozygosity compared to SSR markers. However, the more loci considered, the less variable the estimates of genome-wide heterozygosity and the better the precision and accuracy; thus, achieving a greater number of loci is more realistic with SNPs than with SSR markers [60] because of the high-throughput that is easily attained with SNPs. However, the majority of SNPs in polyploids do not segregate as SDMs, as reported previously [53] for the complex polyploid sugarcane genome, in which only 30,5% of the SNPs evaluated were SDMs. Moreover, most of the SNPs identified in polyploids are duplicated in the genome and in tandem. Allelic dosage and genetic copies are two important features of polyploid mapping that are confounded with one another, as also noted previously [61]. It is fundamentally necessary that the genetic copies and allelic dosage within a locus can be differentiated, and this need for differentiation is one of the most limiting factors in genetic mapping in plants, particularly in polyploid plants. Along with appropriate statistical approaches for polyploid mapping, the differentiation is the only method to obtain more representative and adequate genetic maps for polyploids.

It is important to note that the type C bands allow the integration of the parents’ maps, thereby enabling advances relative to the pseudo-testcross approach [31, 62]. Although type C bands are less informative, they allow for the establishment of indirect linkages between the bands from each parent (type D1 and D2 bands), thereby leading to the construction of an integrated genetic map [63]. Linkages between type D1 bands (from parent H031) and type D2 bands (from parent cv. BRS Tupi) through type C bands were observed in nineteen LGs: LG4, LG6, LG10, LG11, LG13, LG15, LG16, LG17, LG22, LG24, LG26, LG29, LG38, LG41, LG43, LG44, LG45, LG47, and LG49. From these, LGs 13, 17 and 22 presented type C, type D1 and type D2 linked bands, representing a significant number of integrated LGs, whereas for sugarcane, a polyploid grass, only one integrated LG was identified [30]. Among the other LGs, 16 presented only type D1 bands, and 14 presented only type D2 bands.

Karyotype studies in *U*. *humidicola* revealed chromosomes between 1.85 and 3.78 μm long in a hexaploid accession [13]. Compared to the chromosomes of wheat (between 8.4 and 13.8 μm long) [64] and *Bromus riparius* (between 5.5 and 6.8 μm long) [65], both of which are grasses, *U*. *humidicola* has small chromosomes. For other species, a strong correlation between the physical size of the chromosome and the number of markers per LG has been observed [66, 67]. Therefore, the results obtained from the linkage map, with LGs with an average size of 34.7 cM, are consistent with cytogenetic studies.

The identification of eight HGs is similar to the expected six HGs because the parents presented 2n = 6x = 36 chromosomes; this result is reasonable for the first genetic map for the species. Previous studies of polyploid grasses that used the same methodology [68, 69] reached the expected number of HGs, but they used a greater number of markers.

The power to detect linkages between markers in the repulsion phase in polyploids depends on the size of the mapping population, on the ploidy level and on the behavior of the chromosomes in meiosis pairing [24]. When chromosomes pair randomly, as in the case for autopolyploids, the power to detect the linkages between markers in repulsion with 80% power for all possible allelic configurations [19]. The present work evaluated 279 F_1_ progenies and, therefore, considering the difficulty in detecting SDMs in polyploids, linkages in repulsion were easily detected with high and significant LOD scores (Table 4), which reinforces our evidence for disomy.

The origins of the polyploidy presented in this species remain unknown; however, evidence for allopolyploidy in other *Urochloa* species has been presented previously [14, 16, 70]. Moreover, little is known about either the genome structure or inheritance in *U*. *humidicola*, leading to mapping difficulties. Distinguishing among polyploidy types can be difficult; thus, cytogenetic, morphologic and genetic studies, in addition to information regarding fertility, may be necessary to determine whether the species is autopolyploid, segmental allopolyploid, true allopolyploid or autoallopolyploid [71]. Moreover, allopolyploidy and autopolyploidy are extremes on a continuum between bi- and multivalent chromosomal associations and di- and polysomic loci segregation, and several species, mainly those with recent hybridization events, present a combination of these associations and segregations.

**Fig 5:**
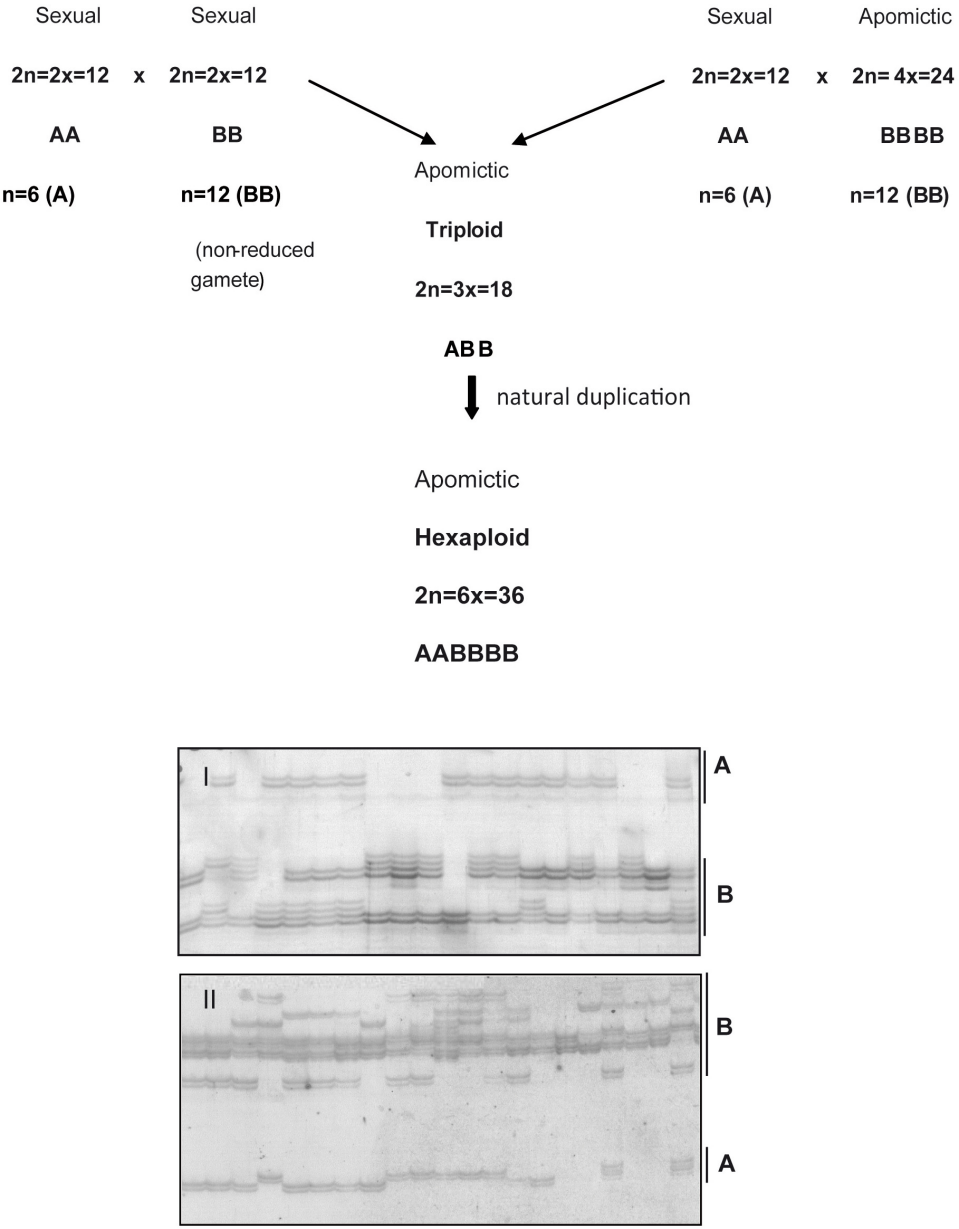
Suggested origin of *U*. *humidicola*. Illustrative scheme of the origin of the hexaploid *Urochloa humidicola*, based on cytogenetic and molecular data. The two different genomes are indicated as A and B. The figures represent the amplification profiles of two microsatellite loci (I. BhUNICAMP010 and II. BhUNICAMP037) and the corresponding amplification regions of genomes A and B.

The observed types of chromosome associations at diakinesis, the presence of two nucleoli in some meiocytes, the different genome sizes and their preferential allocation in the first metaphase plate and asynchronous chromosomal migration to the poles during anaphases, along with the occurrence of di- and polysomic loci, are evidence of allopolyploidy [71, 72]. A similar approach using cytological and segregation behavior suggests an allopolyploid origin for *Salix* species [73]. Our data suggest that the genotypes H031 and cv. BRS Tupi of *U*. *humidicola* are recent natural allopolyploids and that these accessions most likely originated from a cross between a diploid sexual female (2n = 2x = 12, genome A) and a tetraploid apomictic male (2n = 4x = 24, genome B) that, after meiosis, gave rise to a triploid (2n = 3x = 18, ABB). The triploid could have also originated from the cross of two diploid parents (2n = 2x = 12), one contributing a reduced gamete (n = 6, genome A) and the other contributing an unreduced gamete (n = 12, genome B), which has already been described for three *Urochloa* species, including *U*. *humidicola* [18] (Fig 5). After natural chromosome duplication, an allohexaploid (2n = 6x = 36, AABBBB) was formed. The basic chromosome number x = 6 was reported for *U*. *humidicola* [8, 9, 10] and also for *U*. *dictyoneura* [74], a species closely related to *U*. *humidicola*.

Asexuality should lead to phenomena such as genetic and chromosomal mutation accumulation [75] and partial or full linkage disequilibrium, which together influence chromosomal pairing and marker segregation. Apomictic plants are known to not recombine and, consequently, accumulate mutations in their genomes during evolution [75]. However, apomixis in *U*. *humidicola* is facultative, which leads to a percentage of sexuality that can avoid mutation accumulation through the action of natural selection, as observed for the hexaploid *Ranunculus auricomus* [75].

The predominance of meiocytes with bivalents and tetravalents indicates that the B genome allowed the formation of tetravalents. However, the occurrence of hexavalents, although in low frequency, reveals that the A genome exhibits a certain homeology with the B genome, ensuring some chromosome associations between them, which was also observed in the genetic map.

The results of studies of several polyploid accessions of different species in the genus *Urochloa* indicate that only one nucleolus is found in meiocytes with evidence of paleopolyploidy [70, 74, 76, 77, 78]. However, in accessions with evidence of recent natural hybridization, which is also observed in other *U*. *humidicola* accessions [8, 9, 10], two nucleoli can be found in some meiocytes. Although there is considerable evidence for nucleolus dominance in hybrids [79, 80, 81], the presence of two nucleoli in some meiocytes of the *U*. *humidicola* hybrids analyzed (Fig 2), in addition to other cytological findings, reinforces the assumptions that the parental accessions H031 and cv. BRS Tupi are natural allopolyploids and that the genomes have not yet coordinated to organize a single nucleolus. In the majority of meiocytes with two nucleoli, one nucleolus is smaller than the other.

The cytological analyses of meiocytes at pachytene in the hybrids suggest that genomes A and B are of different sizes. Genome A appears to have small chromosomes with more regions of heterochromatin (Fig 3a), as corroborated by the observation of six small bivalents in diakinesis (Fig 3b). This small genome was also detected in metaphase I when it exhibited a preferential allocation in the metaphase plate. Differences in the genome sizes in *B*. *humidicola* were detected via flow cytometry [11]. The six small bivalents exhibited a tendency to occupy a lateral position in the metaphase plate (Fig 3c) and occasionally remained isolated (Fig 3d). The separation of genomes in the metaphase plate was also observed in an interspecific hybrid between *U*. *ruziziensis* and *U*. *brizantha* [82].

**Fig 6:**
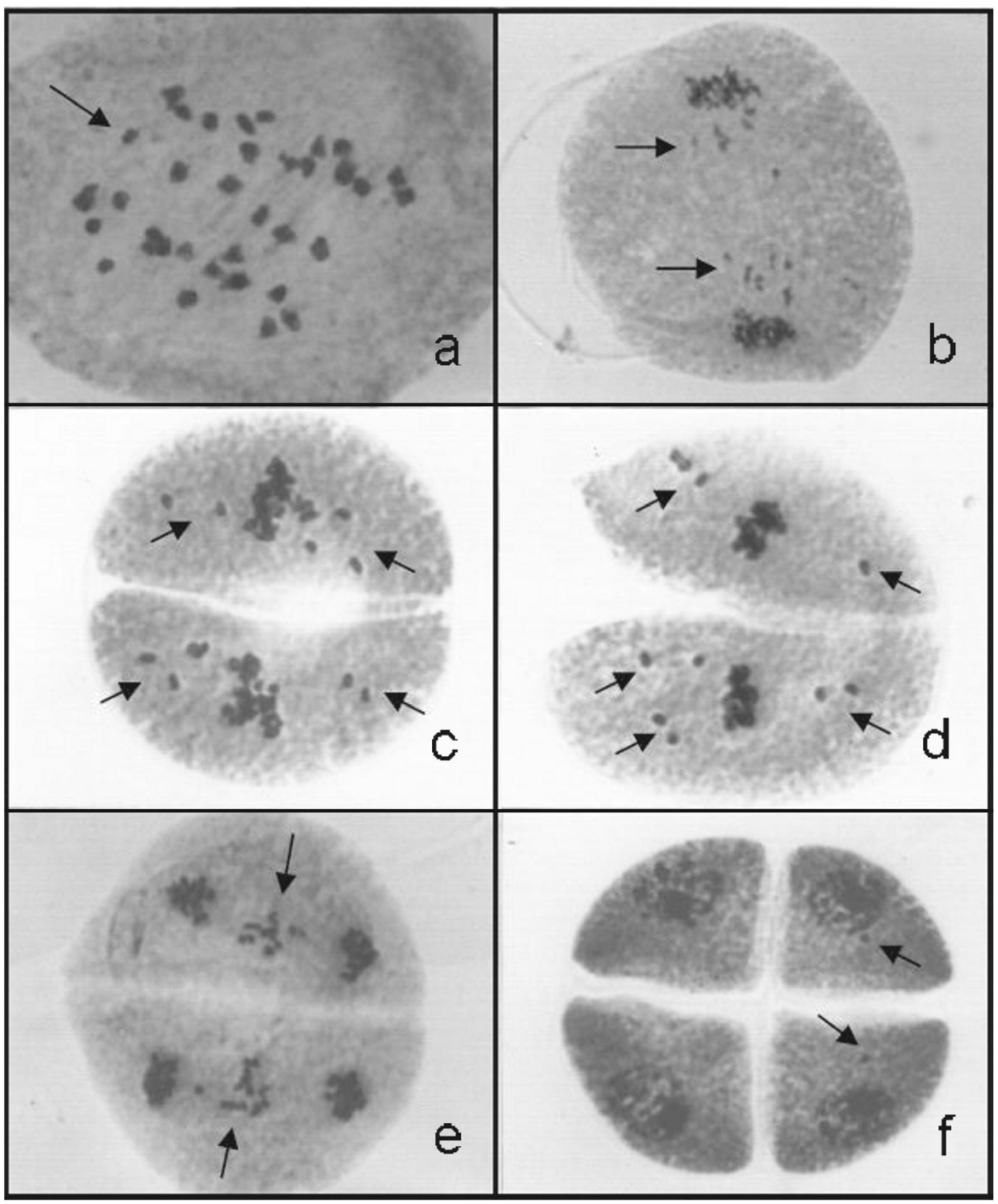
Meiotic behavior in the *U*. *humidicola* hybrids. Meiotic behavior evaluated in the hybrids (2n = 36) listed in Tables 1 and 2. a) Meiocyte in anaphase I exhibiting 12 segregated chromosomes of genome B and six small bivalents of genome A, with late disjunction, in metaphase I (arrow). b) Anaphase I with the laggard chromosomes of genome A (arrows). c, d) Metaphase II (genome B) with six scattered chromosomes of genome A (arrows). e) Late anaphase II with genome A in the metaphase plate (arrow). f) Tetrad with small micronuclei in two microspores (arrows).

In the hybrids, typical allopolyploid behavior was observed. In anaphase I, when genome B was migrating to the poles, with 12 segregated chromosomes at each pole, six small bivalents from genome A remained at the metaphase plate (Fig 6a). When genome B reached the poles, genome A was in anaphase I (Fig 6b), although genome A reached the poles in time to be included in the nuclei with genome B. Only a few chromosomes remained outside the nuclei, forming micronuclei. In the second division, the meiotic behavior was the same, with genome A lagging relative to genome B. In the second division, the genomes were more asynchronous among the phases than in the first division. Fig 6c and 6d show the six chromosomes of genome A scattered within the cytoplasm, whereas the 12 chromosomes of genome B are in metaphase II. Genome A remained a laggard in anaphase II (Fig 6e). However, the majority of the chromosomes of genome A were included in the nuclei, and only a few small micronuclei were observed in the tetrads (Fig 6f). The mean percentage of meiotic abnormalities among the hybrids ranged from 20.72 to 81.40%.

Asynchrony during microsporogenesis, similar to that observed in the present hybrids, has been reported in polyploid accessions of *U*. *brizantha* [82] and in accessions of *U*. *humidicola* with an odd level of ploidy [8, 9, 10], thereby suggesting recent natural hybridization events. Asynchrony in the *U*. *humidicola* hybrids also suggests that the genitors are allopolyploids and that their parental species do not have the same meiotic rhythm.

By observing the SSR amplification pattern, we found that more than half (50.6%) of the loci amplified two sets of bands, thus corroborating the presence of two different genomes suggested by the meiotic analyses in this study and in a study of the genetic diversity of the species’ germplasm [43]. More bands in a set of bands observed for certain SSR loci (Fig 5) suggest that there are more copies of the alleles in genome B, which would suggest higher dosages in this genome for the observed loci. Moreover, loci with disomic inheritance from autopolyploids, which are characteristic of allopolyploidy, in addition to loci with polysomic inheritance, were observed, thus suggesting a recent allopolyploid origin. This hypothesis would explain the difficulty of grouping the LGs into HGs because the chromosome set would be formed by both homologous and homeologous chromosomes. In addition, the exclusive use of SDMs would be a limiting factor in the detection of HGs.

The results of the identification of the mode of reproduction of the hybrids corroborate the hypothesis that a single dominant gene controls the aposporic apomixis [2]. Apospory was mapped to LG02, as described for *Paspalum notatum* [29] and for *Brachiaria* sp. [6], which corroborates studies of this characteristic in other grasses that reveal a single ASGR that does not undergo recombination [35, 36]. More markers are required to saturate the region linked to apospory, and we suggest the use of functional markers linked to genes related to this trait, such as SCAR, TRAP and EST-SSR markers.

This study describes the development of a linkage map that provides important information about the genome of the species, and it can be used in further studies of *Urochloa* spp. Moreover, it represents the first step towards a high-coverage map and a QTL map, which are significant for breeding programs focusing on apomictic species. With 1702.82 cM of the genome covered, the hybrids used in this study are well suited for linkage analyses. The addition of more markers to the map, such as the newly developed SSRs presented in a previous study [83] and markers with higher dosages, would increase the resolution, and map coverage studies are currently being conducted by our research group. Complementary cytogenetic studies, such as in situ hybridization, would shed light on the evolution of the genome of this species. The heterozygous parents are also segregant for their growth habit, tillering intensity, leaf width and productivity, thereby allowing future QTL studies to target these characteristics.

## Conclusions and Perspectives

This study suggests an allopolyploid origin for *U*. *humidicola* based on meiotic analyses and the construction of the first genetic map for this species. Moreover, it describes a new set of SSR markers for *U*. *humidicola* and maps the apo-locus to a single linkage group. The inclusion of multiple-dose markers and map saturation are further steps to map apomixis and other characteristics of interest.

## Materials and Methods

We confirm that no specific permits were required for the described field studies. The collections were performed on the research institution Embrapa Beef Cattle, and no specific permission was required for these locations and activities. We confirm that this study did not involve endangered or protected species.

### Plant material and mapping population

As part of the *Urochloa* breeding program of the Brazilian Agricultural Research Corporation (Embrapa) Beef Cattle (EBC), Campo Grande/MS, an intraspecific cross was made: H031 (sexual accession) x *U*. *humidicola* cv. BRS Tupi (apomictic cultivar, used as pollen donor); both parents were hexaploid (2n = 6x = 36). A full-sib progeny of 361 F_1_ individuals was obtained [44], from which 279 hybrids were identified using RAPD markers [42, 84]. These hybrids were used for the cytogenetic analysis and construction of the genetic linkage map. These progeny have been maintained in the field at EBC since 2005. Leaf samples from each hybrid and their parents were collected, frozen and dried prior to extracting the genomic DNA as described previously [3].

### Meiotic analysis

Inflorescences were collected from both parents and 45 hybrids for meiotic studies according to a method described previously [44]. The inflorescences were fixed in a mixture of ethanol-chloroform-propionic acid (6:3:2, v/v/v) for 24 h and then stored under refrigeration in 70% ethanol. Microsporocytes were obtained by squashing the anthers in a drop of 1% propionic carmine. Photomicrographs were obtained with a Wild Leitz microscope using Kodak Imagelink—HQ, ISO 25 black-and-white film.

Chromosome associations were examined in 20 meiocytes at diakinesis under light microscopy. The types of chromosome associations were expressed as percentages.

### Identification of the mode of reproduction

The mode of reproduction of each individual was previously determined by [41, 45, 46] through an examination of embryo sacs using interference contrast microscopy on methylsalicilate-cleared ovaries according to a method described previously [85]. A total of 270 hybrids from the mapping population were analyzed through the dissection of 60 ovules/hybrid and visualization of at least 50 cleared ovules/hybrid. A chi-square test was performed with these progeny to verify whether the genetic segregation for apomictic to sexual plants fit the expected model for monogenic inheritance.

### Microsatellite marker development

New microsatellite markers were developed for *U*. *humidicola* from a previously constructed microsatellite-enriched library [3] according to methods described previously [86]. Polymerase chain reaction (PCR) was performed as previously described [3]. The amplification products were resolved by electrophoresis in 3% agarose gels prior to vertical electrophoresis in 6% denaturing polyacrylamide gels, which were then silver-stained as previously described [87]. The product sizes were determined by comparison with a 10-bp DNA ladder (Invitrogen, Carlsbad, CA).

### Analysis of microsatellite markers

Along with the new microsatellites, markers that had been previously identified [3, 86] were used in this study (S2 Table). An evaluation was previously performed on the parents of the cross and on six hybrids to verify the polymorphism of the loci. Each allele was evaluated independently as a dominant marker because of the polyploid nature of the genotypes. Accordingly, the data were scored based on the presence (1) or absence (0) of a band for each of the 279 hybrids and the parents.

### Notation for markers

Microsatellite markers were identified with the acronym Bh (from the species) and the name of the institution at which they were developed (UNICAMP), followed by sequential numbers from markers previously developed [3, 86].

For the construction of the linkage map, the bands were named with the acronym Bhx-y-z, in which “x” corresponds to the number of the locus, “y” corresponds to the amplified band in locus “x”, and “z” corresponds to the origin of the polymorphism of the parent, the last being designated according to a previously described method [88]. “D1” corresponds to bands that were heterozygous for H031 and homozygous for *U*. *humidicola* cv. BRS Tupi, thus following the “ao x oo” cross configuration; “D2” corresponds to bands that were homozygous for H031 and heterozygous for *U*. *humidicola* cv. BRS Tupi (“oo x ao” cross configuration); and “C” corresponds to bands that were heterozygous in both parents (“ao x ao” cross configuration). This notation was proposed previously [50]. The single-dose allele (*simplex*) is represented by “a” and is dominant to the null allele (*nulliplex*), which is represented by “o”. The notations “D1” and “D2” refer to the bands in a *testcross* configuration between the parents and segregate in a 1:1 ratio, and “C” refers to heterozygous loci in both parents in a 3:1 ratio, with the same genotype in both parents. The homology groups (HGs) are identified by Roman numerals.

### Marker segregation

Each allele was evaluated independently as a dominant marker based on its presence (1) or absence (0) in each of the 279 hybrids and their parents. Because of the polyploid nature of the species, only SDMs [88] were used for linkage map construction. For the identification of bands that presented 1:1 or 3:1 ratios, as expected for SDMs found in only one parent (*testcross* configuration) and SDMs found in both parents, respectively, a chi-square (X^2^) test was used. Bands with segregation distortion were not considered. To avoid Type I error, the Bonferroni correction for multiple tests was used, considering 0.05 as the overall significance level. Bands that deviated from the expected segregation ratio after the Bonferroni correction were not included in the linkage analysis because they may indicate loci with higher doses.

### Linkage analysis

Linkage analysis and map construction were performed using the OneMap software package [89], which was developed based on a multipoint approach [88] and recently included a multipoint approach based on Markov chains [90]. The two-point analysis was performed using LOD scores of 5 and 20 cM [91] for the recombination fraction. For an LG with up to six bands, linked markers were ordered using the *compare* command, in which the different possible orders of bands were compared based on their respective likelihoods. For larger groups, the *compare* command became computationally unfeasible, and the *order* command was used. The results were verified using the *ripple* command when necessary.

The LGs were designated as “LGx”, in which “x” is the number of the LG, followed by the type of bands present. If the LG presented only type D1 or D2 bands, it was classified and named as LGx_D1 or LGx_D2, respectively. If the LG also presented type C bands, it was classified and named as LGx_I to indicate that it was integrated because the type C bands are heterozygous in both parents and allow linkage between type D1 and D2 bands (Fig 4).

### Homology groups

Putative homology groups (HGs) were determined based on a previous study [22]. LGs were assembled into HGs when at least two bands derived from the same locus were shared, with the locus being defined as the primer pair that flanks the microsatellite.

## Supporting Information

S1 Table New microsatellite markers developed for *Urochloa humidicola* (syn. *Brachiaria humidicola*) and their usage for the linkage map construction. (XLSX)

S2 Table Published polymorphic microsatellite markers used in this study. (XLSX)

S3 Table Genotyping data from the 102 polymorphic SSR loci used for the genetic map construction. Presence of bands is indicated by 1 and absence of bands is indicated by 0. Loci are named according to the notation described in the manuscript. Bands included in the linkage map are highlighted in grey.
(XLSX)

S4 Table Linkage phase diagram of each band in both genitors of the mapping population at each LG. The configuration ao x ao indicates a type C band, ao x oo indicates a type D1 band, and oo x ao indicates a type D2 band. Linkages in repulsion are indicated in bold text.
(DOCX)

S5 Table Mode of reproduction of the mapping population and its genitors. Mode of reproduction of each genotype studied. APO: apomictic, SEX: sexual
(XLS)

S1 Fig Amplification profiles of certain SSR loci used in the study. Allelic profiles of the genitors (P_1_ refers to H031 and P_2_ refers to cv. BRS Tupi) and several hybrids for the loci BhUNICAMP058 (A), BhUNICAMP038 (B), BhUNICAMP171 (C), BhUNICAMP029 (D), BhUNICAMP164 (E), BhUNICAMP003 (F), BhUNICAMP010 (G) and BhUNICAMP037 (H). The lack of amplification in P_1_ (H031) is represented in (A), (B), (C) and (D), with examples of loci with disomic inheritance. The amplification of two different sets of bands is indicated in (E), (F), (G) and (H), in which one genomic region presents more alleles from the same locus than the other, with polysomic inheritance of the locus. The fragment sizes in terms of the numbers of base pairs are indicated on the right sides of the figures.
(TIF)
