# Supplementary material for: Evidence of Allopolyploidy in Urochloa humidicola Based on Cytological Analysis and Genetic Linkage Mapping
Source: PLoS One. 2016 Apr 22;11(4):e0153764. doi: 10.1371/journal.pone.0153764 (PMC4841517; doi:10.1371/journal.pone.0153764)
Supplement: S4 Table — The configuration ao x ao indicates a type C band, ao x oo indicates a type D1 band, and oo x ao indicates a type D2 band. Linkages in repulsion are indicated in bold text. (DOCX) [file pone.0153764.s004.docx]

| **Supporting Table 4.** Linkage phase diagram of each band in both genitors of the mapping population, at each LG. The configuration ao x ao indicates a type C band, ao x oo indicates a type D1 band, and oo x ao indicates a type D2 band. Linkages in repulsion are indicated in blue. | | | | | | | | |
| --- | --- | --- | --- | --- | --- | --- | --- | --- |
|  |  |  |  |  |  |  |  |  |
|  |  |  |  |  |  |  |  |  |
| LG1 |  |  |  |  | LG7 |  |  |  |
| Bands | Position (cM) | Parent 1 | Parent 2 |  | Bands | Position (cM) | Parent 1 | Parent 2 |
| Bh026.a.D2 | 0 | o \| \| o | a \| \| o |  | Bh171.a.D2 | 0 | o \| \| o | a \| \| o |
| Bh015.b.D2 | 16.58 | o \| \| o | a \| \| o |  | Bh020.c.D2 | 13.31 | o \| \| o | a \| \| o |
| log-likelihood: | -314.2973 |  |  |  | Bh192.e.D2 | 34.32 | o \| \| o | a \| \| o |
|  |  |  |  |  | log-likelihood: | -438.4533 |  |  |
| LG2 |  |  |  |  |  |  |  |  |
| Bands | Position (cM) | Parent 1 | Parent 2 |  | LG8 |  |  |  |
| Bh146.a.D2 | 0 | o \| \| o | a \| \| o |  | Bands | Position (cM) | Parent 1 | Parent 2 |
| Bh026.e.D2 | 10.85 | o \| \| o | a \| \| o |  | Bh140.a.D2 | 0 | o \| \| o | a \| \| o |
| Bh027.c.D2 | 36.56 | o \| \| o | a \| \| o |  | Bh140.b.D2 | 1.08 | o \| \| o | a \| \| o |
| log-likelihood: | -431.9428 |  |  |  | Bh140.c.D2 | 7.23 | o \| \| o | a \| \| o |
|  |  |  |  |  | log-likelihood: | -273.2399 |  |  |
| LG3 |  |  |  |  |  |  |  |  |
| Bands | Position (cM) | Parent 1 | Parent 2 |  | LG9 |  |  |  |
| Bh015.a.D1 | 0 | a \| \| o | o \| \| o |  | Bands | Position (cM) | Parent 1 | Parent 2 |
| Bh147.b.D1 | 12.72 | a \| \| o | o \| \| o |  | Bh140.e.D1 | 0 | a \| \| o | o \| \| o |
| log-likelihood: | -296.0028 |  |  |  | Bh150.c.D1 | 15.26 | a \| \| o | o \| \| o |
|  |  |  |  |  | Bh145.b.D1 | 22.16 | a \| \| o | o \| \| o |
| LG4 |  |  |  |  | log-likelihood: | -374.4686 |  |  |
| Bands | Position (cM) | Parent 1 | Parent 2 |  |  |  |  |  |
| Bh015.f.D2 | 0 | o \| \| o | a \| \| o |  | LG10 |  |  |  |
| Bh035.b.C | 3.34 | o \| \| a | a \| \| o |  | Bands | Position (cM) | Parent 1 | Parent 2 |
| Bh193.b.D2 | 22.54 | o \| \| o | a \| \| o |  | Bh008.a.C | 0 | a \| \| o | a \| \| o |
| log-likelihood: | -381.6439 |  |  |  | Bh200.d.D2 | 24.09 | o \| \| o | o \| \| a |
|  |  |  |  |  | log-likelihood: | -306.8086 |  |  |
| LG5 |  |  |  |  |  |  |  |  |
| Bands | Position (cM) | Parent 1 | Parent 2 |  | LG11 |  |  |  |
| Bh144.a.D1 | 0 | a \| \| o | o \| \| o |  | Bands | Position (cM) | Parent 1 | Parent 2 |
| Bh057.f.D1 | 5.11 | a \| \| o | o \| \| o |  | Bh148.e.D2 | 0 | o \| \| o | a \| \| o |
| Bh150.d.D1 | 12.47 | a \| \| o | o \| \| o |  | Bh152.b.C | 10.30 | o \| \| a | a \| \| o |
| Bh155.d.D1 | 26.41 | a \| \| o | o \| \| o |  | log-likelihood: | -315.3752 |  |  |
| log-likelihood: | -431.0567 |  |  |  |  |  |  |  |
|  |  |  |  |  | LG12 |  |  |  |
| LG6 |  |  |  |  | Bands | Position (cM) | Parent 1 | Parent 2 |
| Bands | Position (cM) | Parent 1 | Parent 2 |  | Bh009.e.D2 | 0 | o \| \| o | a \| \| o |
| Bh035.c.D2 | 0 | o \| \| o | a \| \| o |  | Bh178.f.D2 | 22.64 | o \| \| o | a \| \| o |
| Bh047.a.D2 | 25.95 | o \| \| o | a \| \| o |  | Bh052.d.D2 | 41.57 | o \| \| o | a \| \| o |
| Bh201.b.D2 | 47.55 | o \| \| o | a \| \| o |  | log-likelihood: | -461.9235 |  |  |
| Bh193.e.D2 | 60.56 | o \| \| o | a \| \| o |  |  |  |  |  |
| Bh036.c.C | 73.19 | a \| \| o | a \| \| o |  | LG13 |  |  |  |
| Bh037.i.D2 | 81.07 | o \| \| o | a \| \| o |  | Bands | Position (cM) | Parent 1 | Parent 2 |
| Bh035.a.C | 89.29 | a \| \| o | a \| \| o |  | Bh004.a.D2 | 0 | o \| \| o | a \| \| o |
| Bh044.b.C | 111.57 | a \| \| o | a \| \| o |  | Bh168.d.C | 39.07 | a \| \| o | a \| \| o |
| Bh037.h.D2 | 134.95 | o \| \| o | o \| \| a |  | Bh061.c.D1 | 53.85 | a \| \| o | o \| \| o |
| Bh037.a.D2 | 136.39 | o \| \| o | o \| \| a |  | log-likelihood: | -498.4176 |  |  |
| Bh144.f.D2 | 153.35 | o \| \| o | o \| \| a |  |  |  |  |  |
| Bh144.g.D2 | 153.35 | o \| \| o | o \| \| a |  |  |  |  |  |
| log-likelihood: | -1285.663 |  |  |  |  |  |  |  |

| **Supporting Table 4.** cont. | | | | | | | | |
| --- | --- | --- | --- | --- | --- | --- | --- | --- |
| LG14 |  |  |  |  | LG20 |  |  |  |
| Bands | Position (cM) | Parent 1 | Parent 2 |  | Bands | Position (cM) | Parent 1 | Parent 2 |
| Bh004.d.D1 | 0 | a \| \| o | o \| \| o |  | Bh142.a.D2 | 0 | o \| \| o | a \| \| o |
| Bh061.e.D1 | 10.67 | a \| \| o | o \| \| o |  | Bh168.i.D2 | 8.98 | o \| \| o | a \| \| o |
| log-likelihood: | -286.1482 |  |  |  | log-likelihood: | -269.6396 |  |  |
|  |  |  |  |  |  |  |  |  |
| LG15 |  |  |  |  | LG21 |  |  |  |
| Bands | Position (cM) | Parent 1 | Parent 2 |  | Bands | Position (cM) | Parent 1 | Parent 2 |
| Bh010.a.D2 | 0 | o \| \| o | a \| \| o |  | Bh142.f.D1 | 0 | a \| \| o | o \| \| o |
| Bh161.b.D2 | 24.61 | o \| \| o | a \| \| o |  | Bh168.b.D1 | 8.93 | a \| \| o | o \| \| o |
| Bh052.i.D2 | 39.06 | o \| \| o | a \| \| o |  | Bh025.b.D1 | 14.84 | a \| \| o | o \| \| o |
| Bh178.i.D2 | 63.90 | o \| \| o | a \| \| o |  | log-likelihood: | -332.111 |  |  |
| Bh198.c.D2 | 84.42 | o \| \| o | a \| \| o |  |  |  |  |  |
| Bh039.f.C(M442) |  |  |  |  |  |  |  |  |
| log-likelihood: | -726.5146 |  |  |  | LG22 |  |  |  |
|  |  |  |  |  | Bands | Position (cM) | Parent 1 | Parent 2 |
| LG16 |  |  |  |  | Bh151.c.D1 | 0 | a \| \| o | o \| \| o |
| Bands | Position (cM) | Parent 1 | Parent 2 |  | Bh143.c.D1 | 12.09 | a \| \| o | o \| \| o |
| Bh018.e.D2 | 0 | o \| \| o | a \| \| o |  | Bh197.a.C | 31.19 | a \| \| o | a \| \| o |
| Bh036.b.C | 18.22 | a \| \| o | a \| \| o |  | Bh197.c.D2 | 34.05 | o \| \| o | a \| \| o |
| Bh036.e.D2 | 18.22 | o \| \| o | a \| \| o |  | Bh157.b.D2 | 41.87 | o \| \| o | a \| \| o |
| Bh036.a.C | 30.25 | o \| \| a | o \| \| a |  | Bh143.g.D2 | 52.84 | o \| \| o | a \| \| o |
| log-likelihood: | -549.592 |  |  |  | log-likelihood: | -726.8317 |  |  |
|  |  |  |  |  |  |  |  |  |
| LG17 |  |  |  |  | LG23 |  |  |  |
| Bands | Position (cM) | Parent 1 | Parent 2 |  | Bands | Position (cM) | Parent 1 | Parent 2 |
| Bh142.b.D2 | 0 | o \| \| o | a \| \| o |  | Bh170.h.D1 | 0 | a \| \| o | o \| \| o |
| Bh038.a.D2 | 15.83 | o \| \| o | a \| \| o |  | Bh150.b.D1 | 7.68 | a \| \| o | o \| \| o |
| Bh168.e.D2 | 31.44 | o \| \| o | a \| \| o |  | Bh155.f.D1 | 27.18 | a \| \| o | o \| \| o |
| Bh025.e.C | 36.05 | a \| \| o | a \| \| o |  | Bh157.h.D1 | 42.04 | a \| \| o | o \| \| o |
| Bh168.f.D1 | 39.13 | a \| \| o | o \| \| o |  | log-likelihood: | -516.2 |  |  |
| Bh024.a.D1 | 51.12 | a \| \| o | o \| \| o |  |  |  |  |  |
| log-likelihood: | -756.9842 |  |  |  | LG24 |  |  |  |
|  |  |  |  |  | Bands | Position (cM) | Parent 1 | Parent 2 |
| LG18 |  |  |  |  | Bh021.c.D2 | 0 | o \| \| o | a \| \| o |
| Bands | Position (cM) | Parent 1 | Parent 2 |  | Bh198.d.D2 | 24.66 | o \| \| o | a \| \| o |
| Bh024.b.D1 | 0 | a \| \| o | o \| \| o |  | Bh178.h.D2 | 39.02 | o \| \| o | a \| \| o |
| Bh168.a.D1 | 12.57 | a \| \| o | o \| \| o |  | Bh169.c.D2 | 74.50 | o \| \| o | a \| \| o |
| Bh025.a.D1 | 17.12 | a \| \| o | o \| \| o |  | Bh067.c.C | 85.10 | a \| \| o | a \| \| o |
| Bh142.e.D1 | 26.29 | a \| \| o | o \| \| o |  | Bh052.a.D2 | 108.42 | o \| \| o | a \| \| o |
| log-likelihood: | -424.9907 |  |  |  | Bh161.a.D2 | 119.96 | o \| \| o | a \| \| o |
|  |  |  |  |  | log-likelihood: | -928.6311 |  |  |
| LG19 |  |  |  |  |  |  |  |  |
| Bands | Position (cM) | Parent 1 | Parent 2 |  | LG25 |  |  |  |
| Bh016.b.D2 | 0 | o \| \| o | a \| \| o |  | Bands | Position (cM) | Parent 1 | Parent 2 |
| Bh016.a.D2 | 10.30 | o \| \| o | o \| \| a |  | Bh022.a.D2 | 0 | o \| \| o | a \| \| o |
| Bh037.g.D2 | 41.65 | o \| \| o | o \| \| a |  | Bh037.c.D2 | 12.83 | o \| \| o | a \| \| o |
| Bh145.a.D2 | 52.95 | o \| \| o | o \| \| a |  | log-likelihood: | -286.6932 |  |  |
| Bh145.d.D2 | 79.54 | o \| \| o | o \| \| a |  |  |  |  |  |
| log-likelihood: | -674.62 |  |  |  |  |  |  |  |
|  |  |  |  |  |  |  |  |  |
|  |  |  |  |  |  |  |  |  |

| **Supporting Table 4.** cont. | | | | | | | | |
| --- | --- | --- | --- | --- | --- | --- | --- | --- |
| LG26 |  |  |  |  | LG33 |  |  |  |
| Bands | Position (cM) | Parent 1 | Parent 2 |  | Bands | Position (cM) | Parent 1 | Parent 2 |
| Bh031.a.D2 | 0 | o \| \| o | a \| \| o |  | Bh155.j.D1 | 0 | a \| \| o | o \| \| o |
| Bh161.d.D2 | 20.16 | o \| \| o | a \| \| o |  | Bh048.g.D1 | 16.98 | a \| \| o | o \| \| o |
| Bh067.b.C | 37.28 | a \| \| o | a \| \| o |  | Bh047.f.D1 | 33.11 | a \| \| o | o \| \| o |
| log-likelihood: | -448.254 |  |  |  | log-likelihood: | -428.3845 |  |  |
|  |  |  |  |  |  |  |  |  |
| LG27 |  |  |  |  | LG34 |  |  |  |
| Bands | Position (cM) | Parent 1 | Parent 2 |  | Bands | Position (cM) | Parent 1 | Parent 2 |
| Bh146.f.D1 | 0 | a \| \| o | o \| \| o |  | Bh037.b.D1 | 0 | a \| \| o | o \| \| o |
| Bh067.e.D1 | 23.18 | a \| \| o | o \| \| o |  | Bh174.a.D1 | 23.07 | a \| \| o | o \| \| o |
| log-likelihood: | -314.0574 |  |  |  | log-likelihood: | -333.6177 |  |  |
|  |  |  |  |  |  |  |  |  |
| LG28 |  |  |  |  | LG35 |  |  |  |
| Bands | Position (cM) | Parent 1 | Parent 2 |  | Bands | Position (cM) | Parent 1 | Parent 2 |
| Bh155.b.D1 | 0 | a \| \| o | o \| \| o |  | Bh052.e.D1 | 0 | a \| \| o | o \| \| o |
| Bh167.a.D1 | 12.45 | a \| \| o | o \| \| o |  | Bh178.b.D1 | 18.13 | a \| \| o | o \| \| o |
| Bh167.f.D1 | 22.64 | a \| \| o | o \| \| o |  | Bh198.b.D1 | 30.52 | a \| \| o | o \| \| o |
| Bh155.g.D1 | 35.74 | a \| \| o | o \| \| o |  | log-likelihood: | -417.5042 |  |  |
| Bh200.b.D1 | 59.01 | a \| \| o | o \| \| o |  |  |  |  |  |
| log-likelihood: | -630.06 |  |  |  | LG36 |  |  |  |
|  |  |  |  |  | Marcas | Position (cM) | Parent 1 | Parent 2 |
| LG29 |  |  |  |  | Bh052.g.D1 | 0 | a \| \| o | o \| \| o |
| Bands | Position (cM) | Parent 1 | Parent 2 |  | Bh178.c.D1 | 27.04 | a \| \| o | o \| \| o |
| Bh177.a.C | 0 | a \| \| o | a \| \| o |  | Bh198.a.D1 | 38.98 | a \| \| o | o \| \| o |
| Bh063.a.D2 | 11.81 | o \| \| o | o \| \| a |  | log-likelihood: | -440.978 |  |  |
| Bh063.b.D2 | 12.57 | o \| \| o | a \| \| o |  |  |  |  |  |
| log-likelihood: | -320.5515 |  |  |  | LG37 |  |  |  |
|  |  |  |  |  | Bands | Position (cM) | Parent 1 | Parent 2 |
| LG30 |  |  |  |  | Bh172.b.D1 | 0 | a \| \| o | o \| \| o |
| Bands | Position (cM) | Parent 1 | Parent 2 |  | Bh176.c.D1 | 17.02 | a \| \| o | o \| \| o |
| Bh048.b.D2 | 0 | o \| \| o | a \| \| o |  | Bh155.h.D1 | 36.92 | a \| \| o | o \| \| o |
| Bh141.h.D2 | 15.59 | o \| \| o | a \| \| o |  | log-likelihood: | -442.4922 |  |  |
| log-likelihood: | -297.3844 |  |  |  |  |  |  |  |
|  |  |  |  |  | LG38 |  |  |  |
| LG31 |  |  |  |  | Bands | Position (cM) | Parent 1 | Parent 2 |
| Bands | Position (cM) | Parent 1 | Parent 2 |  | Bh176.g.D2 | 0 | o \| \| o | a \| \| o |
| Bh048.d.D2 | 0 | o \| \| o | a \| \| o |  | Bh172.e.C | 21.98 | a \| \| o | a \| \| o |
| Bh047.d.D2 | 17.19 | o \| \| o | a \| \| o |  | log-likelihood: | -307.5502 |  |  |
| log-likelihood: | -309.3028 |  |  |  |  |  |  |  |
|  |  |  |  |  | LG39 |  |  |  |
| LG32 |  |  |  |  | Bands | Position (cM) | Parent 1 | Parent 2 |
| Bands | Position (cM) | Parent 1 | Parent 2 |  | Bh176.i.D2 | 0 | o \| \| o | a \| \| o |
| Bh048.e.D2 | 0 | o \| \| o | a \| \| o |  | Bh172.c.D2 | 13.67 | o \| \| o | a \| \| o |
| Bh047.e.D2 | 18.49 | o \| \| o | a \| \| o |  | Bh172.d.D2 | 18.17 | o \| \| o | a \| \| o |
| log-likelihood: | -314.0358 |  |  |  | Bh172.f.D2 | 41.80 | o \| \| o | a \| \| o |
|  |  |  |  |  | log-likelihood: | -483.0891 |  |  |
|  |  |  |  |  |  |  |  |  |
|  |  |  |  |  |  |  |  |  |
|  |  |  |  |  |  |  |  |  |
|  |  |  |  |  |  |  |  |  |

| **Supporting Table 4.** cont. | | | | | | | | |
| --- | --- | --- | --- | --- | --- | --- | --- | --- |
| LG40 |  |  |  |  | LG46 |  |  |  |
| Bands | Position (cM) | Parent 1 | Parent 2 |  | Bands | Position (cM) | Parent 1 | Parent 2 |
| Bh149.d.D1 | 0 | a \| \| o | o \| \| o |  | Bh039.a.D2 | 0 | o \| \| o | a \| \| o |
| Bh051.b.D1 | 26.69 | a \| \| o | o \| \| o |  | Bh039.b.D2 | 20.14 | o \| \| o | o \| \| a |
| log-likelihood: | -310.3862 |  |  |  | log-likelihood: | -321.2515 |  |  |
|  |  |  |  |  |  |  |  |  |
| LG41 |  |  |  |  | LG47 |  |  |  |
| Bands | Position (cM) | Parent 1 | Parent 2 |  | Bands | Position (cM) | Parent 1 | Parent 2 |
| Bh193.a.D2 | 0 | o \| \| o | a \| \| o |  | Bh206.a.C | 0 | a \| \| o | a \| \| o |
| Bh153.c.D2 | 22.04 | o \| \| o | a \| \| o |  | Bh196.c.D2 | 6.91 | o \| \| o | a \| \| o |
| Bh151.b.C | 45.73 | a \| \| o | a \| \| o |  | Bh196.b.D2 | 32.62 | o \| \| o | o \| \| a |
| log-likelihood: | -458.2035 |  |  |  | log-likelihood: | -434.5707 |  |  |
|  |  |  |  |  |  |  |  |  |
| LG42 |  |  |  |  | LG48 |  |  |  |
| Bands | Position (cM) | Parent 1 | Parent 2 |  | Bands | Position (cM) | Parent 1 | Parent 2 |
| Bh155.a.D1 | 0 | a \| \| o | o \| \| o |  | Bh164.b.D2 | 0 | o \| \| o | a \| \| o |
| Bh197.b.D1 | 21.71 | a \| \| o | o \| \| o |  | Bh012.a.D2 | 17.53 | o \| \| o | a \| \| a |
| log-likelihood: | -332.1455 |  |  |  | Bh044.e.D2 | 34.15 | o \| \| o | a \| \| o |
|  |  |  |  |  | Bh0164.a.D2 | 55.36 | o \| \| o | o \| \| a |
| LG43 |  |  |  |  | Bh199.d.D2 | 80.40 | o \| \| o | o \| \| a |
| Bands | Position (cM) | Parent 1 | Parent 2 |  | log-likelihood: | -694.9043 |  |  |
| Bh155.i.D1 | 0 | a \| \| o | o \| \| o |  |  |  |  |  |
| Bh047.g.C | 21.24 | a \| \| o | a \| \| o |  | LG49 |  |  |  |
| log-likelihood: | -335.8448 |  |  |  | Bands | Position (cM) | Parent 1 | Parent 2 |
|  |  |  |  |  | Bh043.b.D1 | 0 | a \| \| o | o \| \| o |
| LG44 |  |  |  |  | Bh034.i.C | 4.08 | a \| \| o | a \| \| o |
| Bands | Position (cM) | Parent 1 | Parent 2 |  | Bh149.e.D1 | 10.31 | a \| \| o | o \| \| o |
| Bh162.a.C | 0 | a \| \| o | a \| \| o |  | log-likelihood: | -367.1639 |  |  |
| Bh202.b.D2 | 24.88 | o \| \| o | a \| \| o |  |  |  |  |  |
| log-likelihood: | -311.4651 |  |  |  |  |  |  |  |
|  |  |  |  |  |  |  |  |  |
| LG45 |  |  |  |  |  |  |  |  |
| Bands | Position (cM) | Parent 1 | Parent 2 |  |  |  |  |  |
| Bh157.a.D1 | 0 | a \| \| o | o \| \| o |  |  |  |  |  |
| Bh199.e.C | 13.95 | a \| \| o | a \| \| o |  |  |  |  |  |
| log-likelihood: | -308.8353 |  |  |  |  |  |  |  |
|  |  |  |  |  |  |  |  |  |
