# Supplementary figures and images for: Evidence of Allopolyploidy in Urochloa humidicola Based on Cytological Analysis and Genetic Linkage Mapping
Source: PLoS One. 2016 Apr 22;11(4):e0153764. doi: 10.1371/journal.pone.0153764 (PMC4841517; doi:10.1371/journal.pone.0153764)

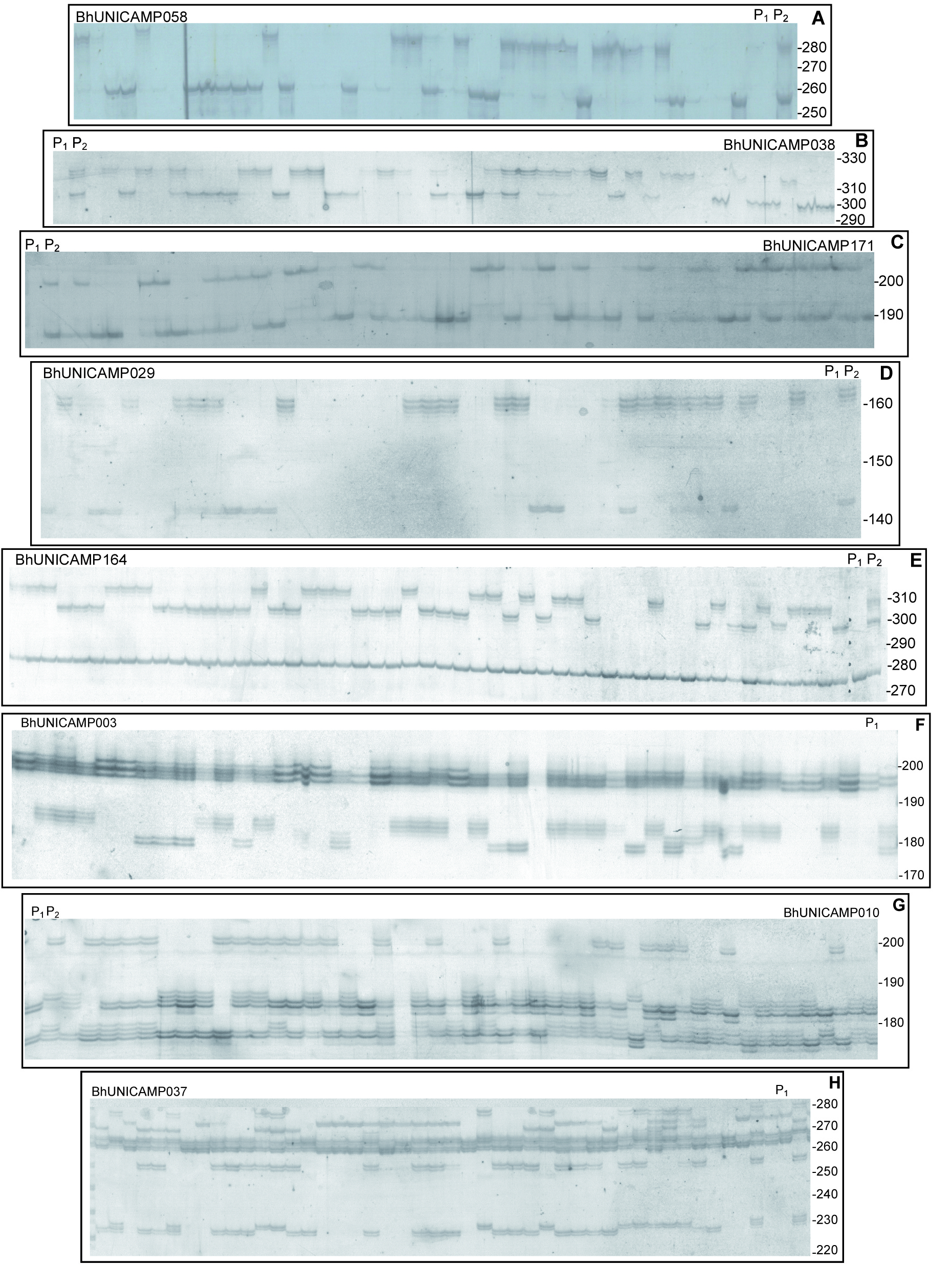

Supplement: S1 Fig — Allelic profiles of the genitors (P1 refers to H031 and P2 refers to cv. BRS Tupi) and several hybrids for the loci BhUNICAMP058 (A), BhUNICAMP038 (B), BhUNICAMP171 (C), BhUNICAMP029 (D), BhUNICAMP164 (E), BhUNICAMP003 (F), BhUNICAMP010 (G) and BhUNICAMP037 (H). The lack of amplification in P1 (H031) is represented in (A), (B), (C) and (D), with examples of loci with disomic inheritance. The amplification of two different sets of bands is indicated in (E), (F), (G) and (H), in which one genomic region presents more alleles from the same locus than the other, with polysomic inheritance of the locus. The fragment sizes in terms of the numbers of base pairs are indicated on the right sides of the figures. (TIF) [file pone.0153764.s006.tif]
